# Matrix metalloproteinase 2 destabilizes Dally-like protein to restrict extracellular Wingless distribution

**DOI:** 10.1091/mbc.E22-09-0434

**Published:** 2025-10-15

**Authors:** Indrayani Waghmare, Patrick S. Page-McCaw, Andrea Page-McCaw

**Affiliations:** ^a^Department of Biological Sciences, University of Massachusetts Lowell, Lowell, MA 01854; ^b^Department of Cell and Developmental Biology, Vanderbilt University School of Medicine, Nashville, TN 37232; ^c^Program in Developmental Biology, Vanderbilt-Ingram Cancer Center, Vanderbilt University School of Medicine, Nashville, TN 37232

## Abstract

Cell-surface glypicans distribute several extracellular ligands, including the Wnts, which are secreted to function at short and long range in a tissue. The *Drosophila* glypican Dally-like protein (Dlp) interacts with Wnts to inhibit short-range Wnt signaling and promote long-range signaling by the *Drosophila* Wnt1, Wingless (Wg). Dlp-dependent long-range Wg distribution in the fly ovary is attenuated by metalloproteinase 2 (Mmp2). Here, we report that Mmp2 destabilizes cell-surface Dlp, causing it to be internalized. Further, after Mmp2 cleavage, Dlp sequesters more Wg, suggesting that cleaved Dlp removes Wg from the extracellular space to limit its availability for signaling. Based on these and our previous results, we propose that coordinated activities of uncleaved and cleaved Dlp regulate proper extracellular Wg distribution. Overall, this study identifies the molecular basis of protease-mediated inhibition of a cell-surface glypican to modulate ligand distribution and function.

## Abstract (executive summary)

- Mmp2 cleaves and destabilizes Dlp on the cell surface, and cleaved Dlp is internalized and likely degraded.
- Cleaved Dlp restricts extracellular Wnt distribution by sequestering more Wnt ligand, whereas uncleaved Dlp promotes extracellular Wnt distribution.
- Relative levels of cleaved and uncleaved Dlp modulate proper extracellular Wnt distribution.

## INTRODUCTION

Wnts form extracellular gradients to activate signaling at short and long range (van den Heuvel *et al.*, 1989; Strigini and Cohen, 2000; Swarup and Verheyen, 2012; Wang and Page-McCaw, 2014; Pani and Goldstein, 2018; Chaudhary *et al.*, 2019; Mii *et al.*, 2021). The extracellular distribution of Wnts is crucial for activating signaling in the target cells and is regulated by cell-surface glypicans (Wang and Page-McCaw, 2014; Beaven and Denholm, 2018; Chaudhary *et al.*, 2019; Tian *et al.*, 2019; McGough *et al.*, 2020; Wang *et al.*, 2021; Waghmare and Page-McCaw, 2022). Dally-like protein (Dlp), the *Drosophila* ortholog of vertebrate glypicans 1, 2, 4, and 6, distributes extracellular Wingless (Wg) and modulates the availability of several Wnts for signaling (Baeg *et al.*, 2001; Baeg *et al.*, 2004; Kirkpatrick *et al.*, 2004; Kreuger *et al.*, 2004; Franch-Marro *et al.*, 2005; Tu *et al*., 2020; Wang and Page-McCaw, 2014; Waghmare *et al.*, 2020). Dlp and Wnts bind each other with low binding affinity (McGough *et al.*, 2020), and the frequent binding and release allow for long-range extracellular Wg distribution to facilitate signaling in cells away from the Wg source while simultaneously restricting ligand availability near the source cells (Yan *et al.*, 2009; McGough *et al.*, 2020; Waghmare and Page-McCaw, 2022). Thus, proper Wg distribution by Dlp depends on cell-surface Dlp levels and is sensitive to changes in Dlp-Wg binding affinity.

The cell surface localization of Dlp is dynamic (Marois *et al.*, 2006; Gallet *et al.*, 2008). Dlp shunts from the apical to basal side of the epithelial cells to distribute Wg (Gallet *et al.*, 2008). Because Wg distribution depends on cell surface Dlp levels, several mechanisms that regulate cell-surface levels of Dlp exist in cells. In the fly germarium, where oogenesis occurs, escort-cell–localized Dlp is negatively regulated by Matrix metalloproteinase2 (Mmp2) to attenuate long-range distribution of Wg secreted from anteriorly located cap cells (CpCs) to posteriorly located follicle stem cells (FSCs) to promote FSC proliferation and proper egg formation (Wang and Page-McCaw, 2014; Waghmare and Page-McCaw, 2018).

In cell culture, Dlp is cleaved in the presence of Mmp2 (Wang and Page-McCaw, 2014). Previously, we proposed a model in which, after cleavage, the Dlp fragment containing the CRD (cysteine-rich domain) was shed into the extracellular space while the C-terminal piece was retained and internalized. The CRD was thought at the time to be the Wg-binding site, and in support of this model, less Wg associated with the cell surface in the presence of Mmp2 (Supplemental Figure S1; Wang and Page-McCaw, 2014). These data were generated using a tagged-Dlp construct that was not tested for functionality, and it was recently shown that Wnts do not bind to CRD in Dlp (McGough *et al.*, 2020). In this study, we revisit the biochemical interaction of Mmp2, Dlp, and Wg using a recently generated tagged Dlp, which is functional (Waghmare *et al.*, 2020).

Using the functional tagged Dlp, we report here that after Mmp2-mediated cleavage, the N-terminal fragment of Dlp remains associated with the C-terminal fragment, and cleaved Dlp is internalized. Cleavage inhibits extracellular Wg distribution and function because the cleaved fragment is capable of sequestering more Wg than its uncleaved form. Based on these observations, we propose that Mmp2 cleavage causes internalization of the Dlp-Wnt complex, negatively regulating Wnt signaling. Proteolytic cleavage–mediated regulation of glypicans is likely an evolutionarily conserved mechanism to modulate biological processes, as previous findings show that proteolytic cleavage of Glypican1 by the metalloproteinase ADAM17 (A Disintegrin And Metalloproteinase17) is important for Glypican1 function to regulate cellular movement, adhesion, and proliferation (Kawahara *et al.*, 2017). However, it is unclear how these biological processes are regulated after Glypican1 cleavage. Here, we identify how proteolytic cleavage of the cell-surface glypican Dlp inhibits Wnt distribution and function.

## RESULTS AND DISCUSSION

### Mmp2 coexpression leads to cleavage and destabilization of Dlp in S2R+ cells

**FIGURE 1:**
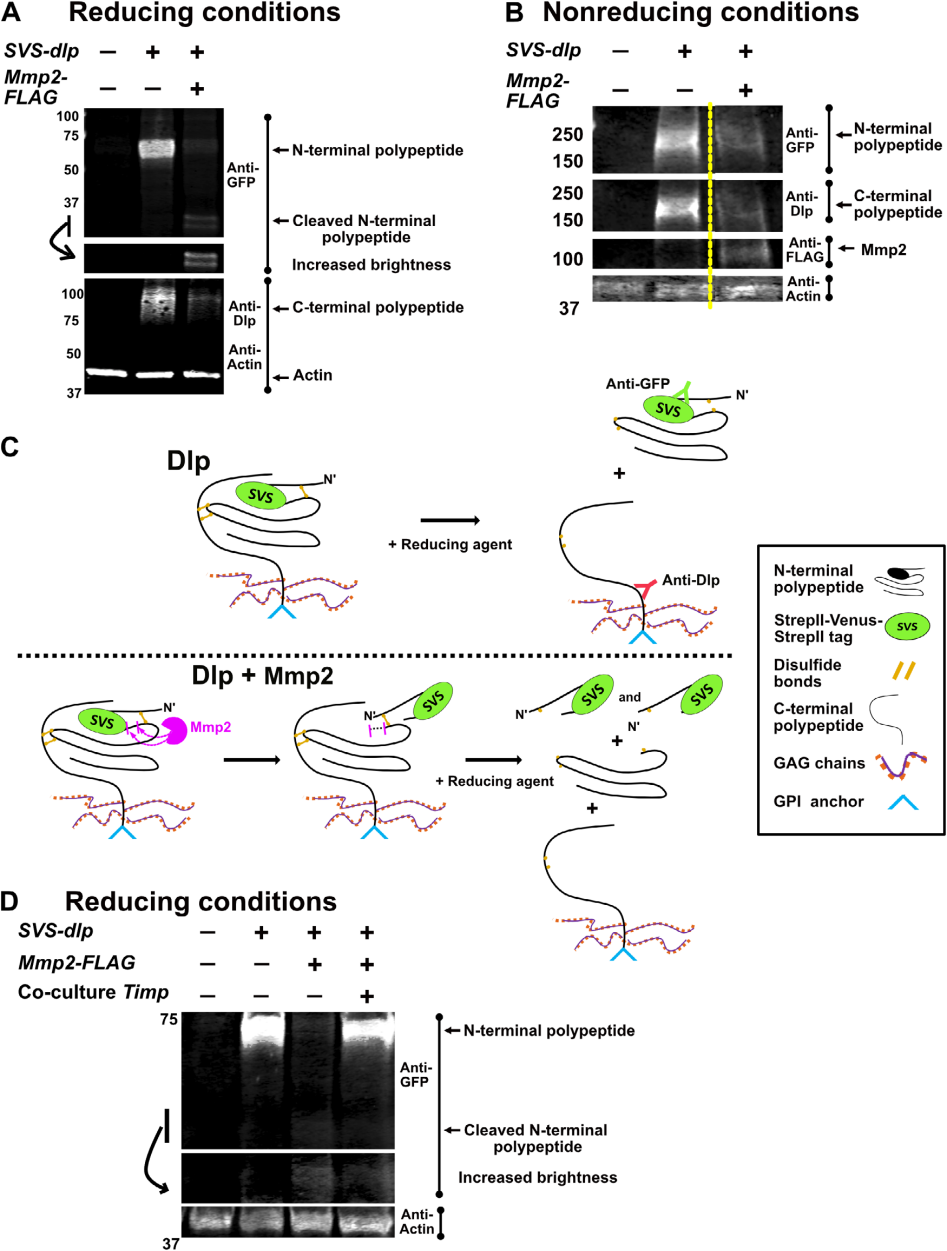
Mmp2 cleaves SVS-Dlp on the cell surface and destabilizes it in S2R+ cells. (A) Western blots of whole cell lysates from cells transfected with the indicated plasmids. Samples were reduced where Dlp N- and C-terminal polypeptides migrate separately. Blots were probed with anti-GFP and anti-Dlp. The ∼30KDa region of the blot probed with anti-GFP is shown with increased brightness. (B) Western blot as in panel A, but samples were not reduced, causing Dlp N and C-terminal polypeptides to remain together and migrate as a smear. Anti-FLAG detects *Mmp2* expression. The dotted yellow line indicates that the intervening lane was excluded. (C) Schematic of mature Dlp and how it migrates under reducing and nonreducing conditions in the absence and presence of Mmp2. (D) Western blot of whole cell lysates from cells expressing an empty control vector, *SVS-dlp,* or *SVS-dlp*+*Mmp2-FLAG* that were cocultured with cells expressing either an empty control vector or *Timp*. Samples were reduced. The blot was probed with anti-GFP. Actin was used as a loading control in A, B, and D.

In transfected S2R+ cells, N-terminally tagged Dlp can be cleaved by Mmp2 (Wang and Page-McCaw, 2014). However, this tagged-Dlp was not tested for function in vivo. To determine if Dlp is indeed cleaved by Mmp2, we expressed a functionally tagged *dlp*, carrying an internal *StrepII-Venus-StrepII* (*SVS*) tag (Waghmare *et al.*, 2020; Delmore and Waghmare, 2025). *SVS-dlp* was expressed with or without *Mmp2* in S2R+ *Drosophila* cells, followed by Western blotting. Dlp is translated as a single polypeptide that is posttranslationally processed in the Golgi to generate N- and C-terminal polypeptides, which are linked by disulfide bonds (Supplemental Figure S2A; Kim *et al.*, 2011). We assessed Dlp first under reducing conditions, which allowed identification of both the N- and C-terminal polypeptides using anti-green fluorescence protein (GFP) and anti-Dlp, respectively (Figure 1A). Anti-GFP recognizes the SVS tag in the N-terminal polypeptide, and anti-Dlp recognizes epitopes on the C-terminal polypeptide. Under reducing conditions, in the absence of Mmp2, the N-terminal Dlp polypeptide was detected. In contrast, in the presence of Mmp2, the amount of Dlp N-terminal polypeptide was greatly decreased (Figure 1A), and low-molecular-weight N-terminal Dlp fragments, representing cleaved N-terminal Dlp polypeptide, were detected (Figure 1A). The presence of two smaller fragments suggests that Dlp has at least two potential cleavage sites within the N-terminal polypeptide. Because our ability to detect cleaved N-terminal fragments relies on the presence of the SVS tag on the cleaved fragments, any additional cleavages that may be occurring within the N-terminal polypeptide will escape detection. The C-terminal polypeptide of Dlp runs as a smear due to the attached glycosaminoglycan (GAG) chains. Similar to the Dlp N-terminal polypeptide, the amount of C-terminal polypeptide was decreased in the presence of Mmp2. However, the apparent molecular weight of the smear was comparable in the presence and absence of Mmp2 (Figure 1A). These data suggest that Mmp2 cleaves Dlp in the N-terminal polypeptide and destabilizes the whole molecule, causing it to be degraded. Although direct cleavage of Dlp by Mmp2 is the simplest possibility, we cannot exclude the possibility that an intermediate protease is activated by Mmp2 to cleave Dlp.

### Dlp retains its N-terminal cleaved fragments after cleavage

Dlp contains seven disulfide bonds (Kim *et al.*, 2011). Analyzing the location of the cysteines using UCSF ChimeraX (Pettersen *et al*., 2021), we tested the possibility that the cleaved products (the cleaved N-terminal Dlp fragments and the rest of the molecule) remain attached to each other through one or more of the disulfide linkages (Supplemental Figure S2A). We tested this possibility by expressing *SVS-dlp* with or without *Mmp2* and assessing the cleavage products under nonreducing conditions (Figure 1B). If the cleaved products remain attached, then Dlp is expected to migrate similarly in the absence or presence of Mmp2. Indeed, Dlp migrated similarly in the absence or presence of Mmp2 under nonreducing conditions, although the intensity of Dlp is decreased in the presence of Mmp2 (Figure 1B). Further, although anti-Dlp and anti-GFP recognize differently sized cleavage products when reduced, they recognize a smear of the same size under nonreducing conditions. Thus, the cleaved Dlp products remain attached, and we reason that cleavage could induce a conformational change, as depicted in Figure 1C.

To determine if any Dlp was lost in the media, we immunoprecipitated the media with anti-GFP beads and analyzed the precipitate (Supplemental Figure S2B). When *SVS-dlp* was coexpressed with either *Mmp2* or catalytically inactive *Mmp2^E258A^*, some uncleaved N-terminal polypeptide was present in the media; additionally, when Mmp2 was active, cleaved N-terminal fragments were also present (Supplemental Figure S2B). Importantly, we never detected the C-terminal polypeptide in the media (not shown). Cell culture media contain significant amounts of cysteine. We propose that the reduction of the Dlp disulfide bond is plausibly due to the presence of cysteine in the media. This cysteine may act to reduce this exposed disulfide bond, releasing the N- and C-terminal polypeptides. Exposed disulfides are known to be labile in media; for example, antibody molecules can be reduced in culture and during harvesting (Chung *et al.*, 2017). Thus, the presence of cysteine in the media might explain the presence of cleaved N-terminal fragments in the media. We conclude that the redox environment controls whether the cleaved fragments remain associated with the cell surface after cleavage. Although it is still possible that some cleaved Dlp fragments are shed extracellularly, we favor the model where cleaved fragments remain disulfide-bonded in vivo (as suggested by our data in Figure 1B), as the in vivo extracellular environment is oxidizing (Ottaviano *et al.*, 2008).

### Mmp2-induced cleavage of Dlp occurs at the cell surface

Both Dlp and Mmp2 localize to the cell surface (Llano *et al.*, 2002; Kim *et al.*, 2011; LaFever *et al.*, 2017), suggesting that Mmp2 cleaves Dlp on the cell surface. However, it is possible that Mmp2 cleaves Dlp in the secretory pathway, as they are coexpressed in the transfected cells in the experiments shown above. Cleavage of Dlp in the secretory pathway would have different implications for the regulation of Wg availability. To investigate whether Mmp2 cleaves Dlp on the cell surface, we cocultured cells expressing both *SVS-dlp* and *Mmp2* with cells expressing *Timp* (Tissue inhibitor of metalloproteinase; Figure 1D). TIMPs are evolutionarily conserved secreted proteins that inhibit metalloproteinase activity by blocking the active site of the MMP (Page-McCaw *et al.*, 2007). When cocultured with control cells, Dlp was cleaved; however, when cocultured with *Timp*-expressing cells, Dlp was not cleaved, and its levels were comparable to control on Western blot (Figure 1D). These results argue that Dlp is cleaved on the cell surface.

### Cleaved Dlp is lost from the cell surface and has an increased rate of internalization

**FIGURE 2:**
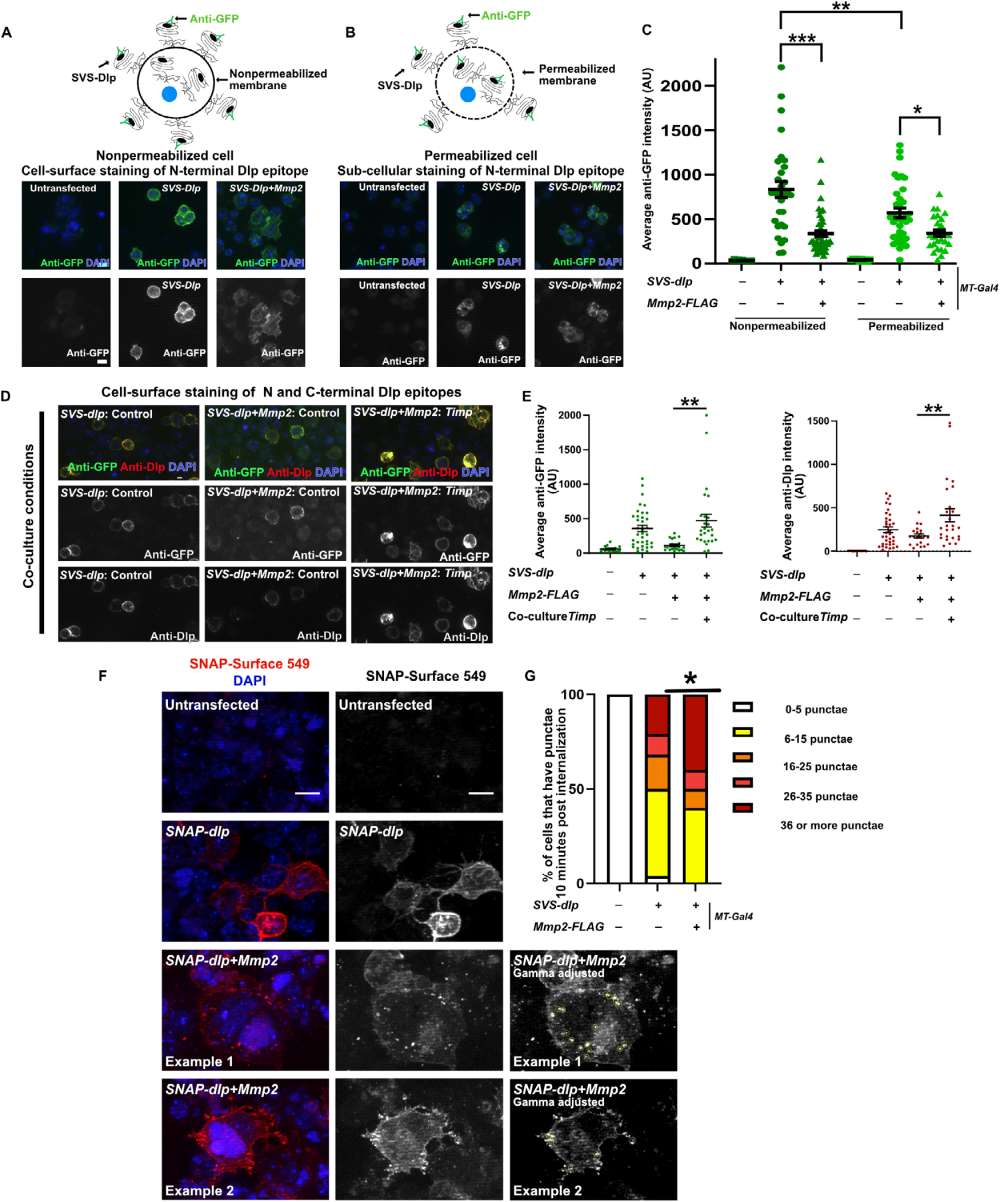
Cell-surface N- and C-terminal SVS-Dlp epitopes are lost in the presence of Mmp2. (A–C) Untransfected (control), *SVS-dlp*, or *UAS-SVS-dlp+Mmp2-FLAG* transfected cells were stained with Anti-GFP staining under nonpermeabilizing (A) and permeabilizing (B) conditions, and quantification of these data is shown in C. (D and E) Cells were cocultured with control or with *Timp*-expressing cells. Anti-GFP and anti-Dlp stainings under nonpermeabilizing conditions are shown in D. Quantification of anti-GFP and anti-Dlp signaling intensity in D is shown in E. (F and G) Untransfected (control), *SNAP-dlp* (*n* = 28), or *UAS-SNAP-dlp+Mmp2-FLAG* (*n* = 20) transfected cells were incubated with cell-impermeable SNAP-Surface 549 fluorogenic substrate (to label cell-surface Dlp) on ice, followed by incubation at 25°C for 10 min to allow internalization. Optical sections of fixed cells were imaged, and the middle slice is shown above to view internalized SNAP-Dlp. Labeling is not detected in untransfected cells but is detected both on the cell surface as well as internalized punctae (dotted yellow circles) in *SNAP-dlp*, or *UAS-SNAP-dlp+Mmp2-FLAG* transfected cells. Panels labeled “Gamma adjusted” indicate that the brightness in these panels was adjusted nonlinearly to facilitate the visualization of punctae. Quantification of internalized labeled Dlp punctae in indicated genotypes is shown in (G). Data shown in C, E, and G were quantified, blinded to the sample identity. Scale bar in A, B, and D: 5 µm. Scale bar in F: 10 µm.

We next tested the effect of Mmp2 cleavage on Dlp's subcellular localization (Figure 2). To monitor cell-surface Dlp, we stained cells expressing *SVS-dlp* or *SVS-dlp*+*Mmp2* with anti-GFP under nonpermeabilizing conditions (Figure 2, A and C). The intensity of anti-GFP was greatly decreased in cells expressing *SVS-dlp*+*Mmp2* compared with cells expressing *SVS-dlp* (Figure 2C), indicating that after cleavage, Dlp is lost from the cell surface. Similarly, the intensity of anti-GFP was greatly decreased in cells expressing *SVS-dlp*+*Mmp2* compared with cells expressing *SVS-dlp* (Figure 2B) when cells were stained under permeabilizing conditions, suggesting that cleaved Dlp is likely degraded. The observed difference in the signal intensities of the N-terminal epitopes detected in our Western blot (Figure 1A) and cell staining data (Figure 2, A–C) can be explained by a combination of different antibodies and different signal detection methods used in these experiments. In agreement with our data in Figure 1D, when cells expressing *SVS-dlp*+*Mmp2* were cocultured with *Timp*-expressing cells, the intensity of both anti-GFP and anti-Dlp staining on the cell surface was increased (Figure 2, D and E). These results indicate that Mmp2 catalytic activity decreases cell-surface Dlp and further confirm that Mmp2 cleaves Dlp on the cell surface.

Our data so far indicate that Mmp2 cleaves Dlp on the cell surface, and the cleaved fragments likely remain associated and are lost from the cell surface. While some cell-surface Dlp is lost in the media, this loss is likely an artifact of cell culture. Therefore, we tested the possibility that cleaved Dlp is lost from the cell surface due to increased internalization; internalization of cell-surface proteins has implications in cell signaling (Di Fiore and von Zastrow, 2014). To directly test whether Dlp was internalized after cleavage, we used SNAP-tagged Dlp, generated by replacing the Venus sequence in SVS tag with SNAP sequence, to track Dlp internalization. SNAP tag allows cell-surface labeling of Dlp when incubated with cell-impermeable SNAP-Surface 549 or SNAP-Surface 488 fluorescent substrates to detect the internalization of fluorescently labeled cell-surface Dlp (Cole, 2013). Cell-surface labeling of Dlp is observed in *SNAP-dlp–*transfected cells, and as expected, most transfected cells do not show intracellular Dlp punctae when cells are fixed and observed immediately after labeling (Supplemental Figure S3, A and D). In contrast, when cells are incubated at 25°C to allow internalization, the number of cells with intracellular Dlp punctae increases at 10 to 20 minutes of incubation at 25°C (Supplemental Figure S3, B–D). These data show that the internalization of Dlp from the cell surface can be reliably observed using *SNAP-dlp*. When cells were cotransfected with *SNAP-dlp* and *Mmp2,* optical sectioning demonstrated that the number of Dlp punctae increased compared with cells transfected with *SNAP-dlp* alone (Figure 2, F and G). Given our evidence that cleaved products remain together unless reduced, and the fact that the extracellular environment in vivo is oxidizing, we expect most of the cleaved Dlp products to remain together after cleavage during internalization of Dlp from the cell surface in vivo. Taken together, our data indicate that Mmp2 cleaves Dlp on the cell surface, but the cleaved fragments remain associated. After cleavage, Dlp is internalized and apparently degraded.

### Mmp2-induced cleavage removes Dlp from the cell surface in vivo

**FIGURE 3:**
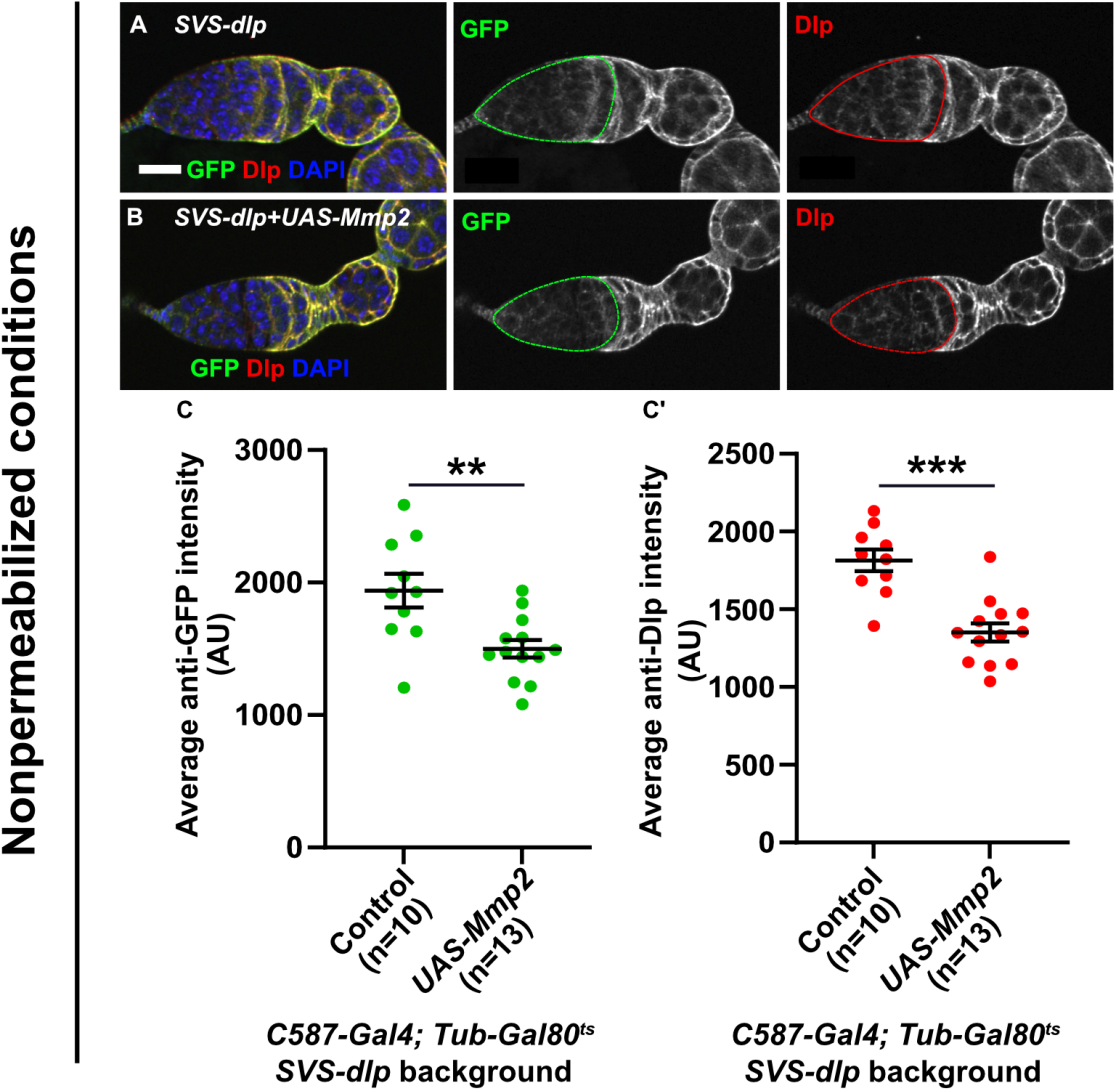
*Mmp2* overexpression decreases cell-surface Dlp. Germaria from control *SVS-dlp* flies (A) or *SVS-dlp* flies conditionally overexpressing *Mmp2* for one day in the escort cells (B) were stained with anti-GFP and anti-Dlp under nonpermeabilizing conditions (A–C). Quantifications of average anti-GFP signal intensity (C) and anti-Dlp signal intensity (C’) are shown. Dotted outline marks the region that was quantified for intensity measurements in sum Z-stacks, although single slices are shown here. Scale bar: 20 µm.

Our data so far are consistent with our previous in vivo data from the fly germarium: in *Mmp2* mutants, the cell surface levels of Dlp increase (Wang and Page-McCaw, 2014). Thus, in vivo, as here in cell culture, Mmp2 removes Dlp from the cell surface. To further confirm this result in vivo, we asked if *Mmp2* overexpression decreases the cell-surface level of Dlp in the fly germarium (Figure 3). We compared cell-surface Dlp staining in germaria isolated from *SVS-dlp* protein-trap flies and *SVS-dlp* flies that conditionally overexpressed *Mmp2* in the escort cells in adulthood. Under nonpermeabilizing conditions, the average signal intensities of both anti-GFP and anti-Dlp stainings were decreased in germaria-overexpressing *Mmp2* compared with control germaria (Figure 3, A–C’).

### Cleaved Dlp sequesters more Wg and other Wnts

**FIGURE 4:**
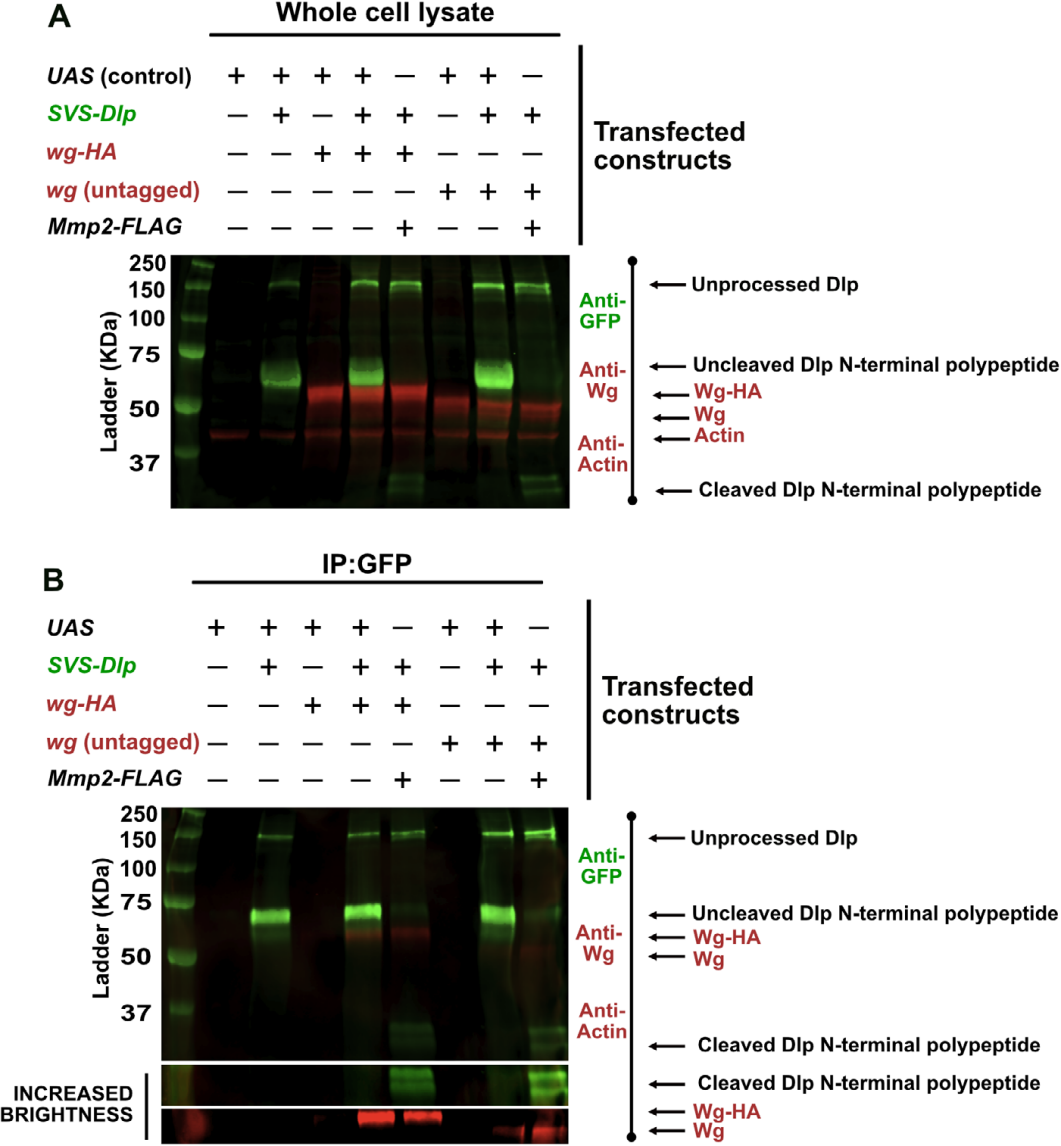
Despite lower levels of cleaved Dlp, both uncleaved and cleaved SVS-Dlp pull down comparable amounts of Wg, suggesting that cleaved Dlp exhibits higher affinity for Wg. Western blots of (A) whole cell lysates or (B) anti-GFP immunoprecipitates were probed with anti-GFP (green) and anti-Wg (red). Actin was used as a loading control in A.

To determine whether Dlp cleavage by Mmp2 affected its interaction with Wg, we expressed *SVS-dlp* and *wg* or *wg-HA* in the absence or presence of *Mmp2* in S2R+ cells, immunoprecipitated Dlp via its SVS tag, and probed for how much Wg was precipitated with Dlp (Figure 4). As expected, the amount of Dlp was significantly decreased in the presence of Mmp2 (Figures 1–4; Supplemental Figures S4 and S5). Unexpectedly, however, this smaller amount of cleaved Dlp was able to pull down comparable amounts of Wg or Wg-HA as uncleaved Dlp, a result that was confirmed in three independent biological replicates (Supplemental Figure S5). Indeed, when Wg was normalized to the amount of Dlp, cleavage increased Wg binding to Dlp by 2.7-fold (median increase for Wg-HA) (Figure 4; Supplemental Figure S5). Similarly, a smaller amount of cleaved Dlp was able to pull down comparable amounts of Wnt2, Wnt4, or Wnt6, all Wnts expressed in the germarium (Waghmare and Page-McCaw, 2018), in three independent biological replicates. (Supplemental Figures S4 and S5). The median increase in Wnt2, Wnt4, and Wnt6 pull down with cleaved Dlp was 6.2-fold, 5.9-fold, and 2.7-fold, respectively (Supplemental Figures S4 and S5). Importantly, in our cell culture experiments, we found no evidence of Wnts being bound to the cleaved or uncleaved Dlp N-terminal polypeptide in the media (data not shown), suggesting that, at least in cell culture, cleaved or uncleaved Dlp N-terminal polypeptides found in the media likely have no implications for Wnt binding or function.

Mmp2 cleavage of Dlp could increase Wg/Wnt binding by increasing binding affinity or by increasing the stoichiometry of binding. Alternatively, Mmp2 could indirectly increase Wnt availability by cleaving other Wnt binding targets. Although the indirect effect of Mmp2 on Wnt may be a possibility in vivo, in our cell culture system, where Wnts are greatly overexpressed, we discount the formal possibility that Mmp2 indirectly increases Wnt availability by cleaving Wnt-sequestering targets. Instead, recent structural studies show that Wnt binds Dlp through the Wnt palmitoleate moiety, which induces a conformational change in Dlp opening a binding a groove between the alpha helices in the N-terminal polypeptide (McGough *et al.*, 2020); these helices are located C-terminal to the putative Mmp2 cleavage sites. An intriguing possibility is that Mmp2 cleavage increases the affinity of Wnt for the groove so that Wnt has a longer occupancy time. Longer occupancy of Wnt on cleaved Dlp, combined with the internalization of Dlp after cleavage, suggests that Mmp2 cleavage of Dlp acts as a Wg sink, removing it from the cell surface. We previously proposed that Mmp2 inhibits Dlp-mediated Wg spreading and activity by removing the Wg-binding site, preventing Wg interaction with cleaved Dlp and allowing its diffusion away from target cells. Our new data, however, suggest a different model: cleaved Dlp inhibits Wg spreading and activity by removing it from play.

### Changes in Mmp2 levels in the fly germarium affect Wg spread

**FIGURE 5:**
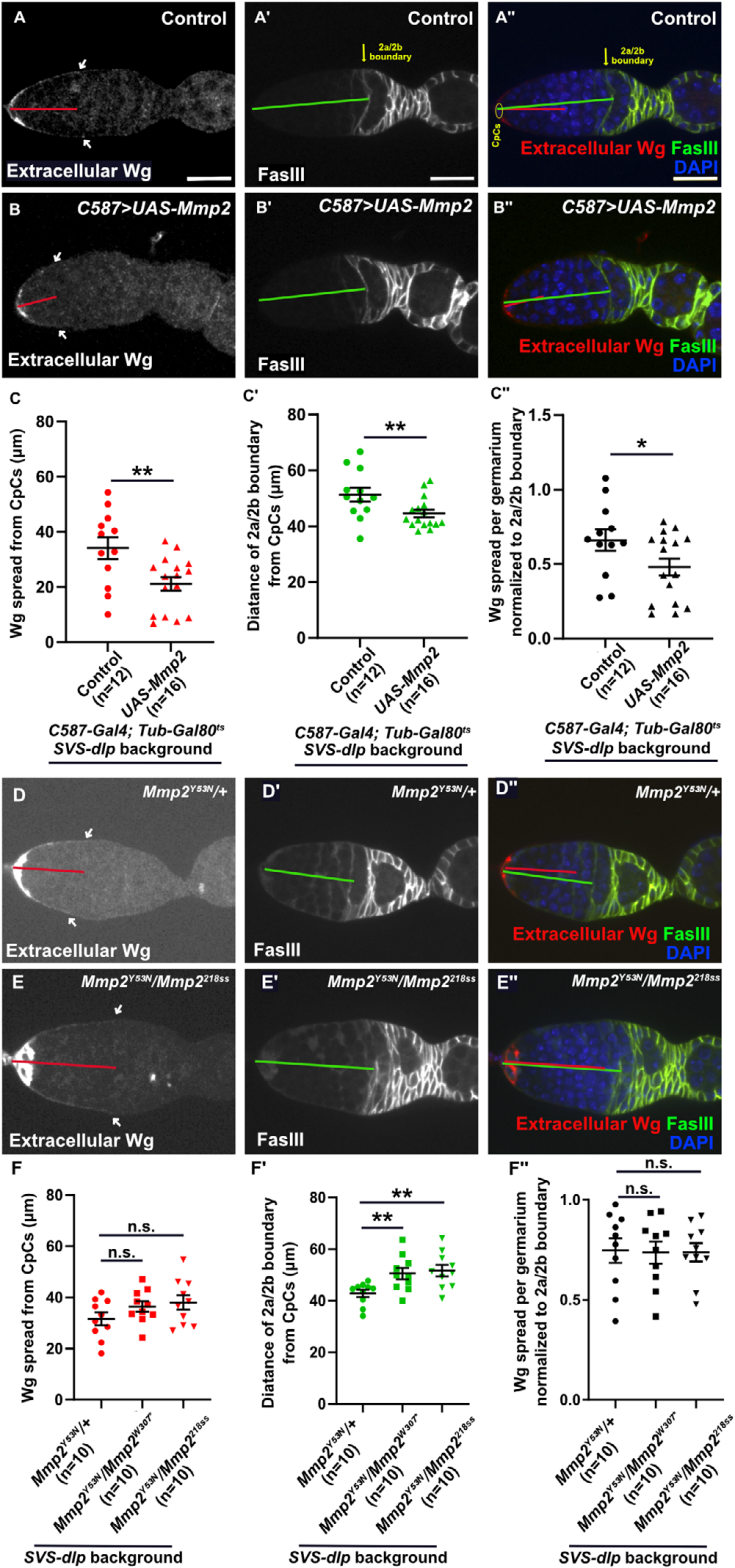
Mmp2 decreases extracellular Wg spreading and germarium length. Extracellular Wg and FasIII stainings in (A–B’’) *Mmp2*-overexpressing germaria or in germaria isolated from (D–E’’) *Mmp2* temperature-sensitive mutants are shown. A single optical section is shown for each germarium. Quantification of (C and F) Wg spread and (C’ and F’) germarium length in *Mmp2*-overexpressing (C–C’’) or mutant (F–F’’). White arrows indicate the extent of Wg spread along the curvature of the germarium. See methods for details. Wg spread was quantified blinded to sample identity. The anterior-posterior (A-P) component of this path, shown as red lines, was measured to quantify Wg spreading. The distance from the cap cells to the 2a/2b boundary (FasIII staining) is indicated by green lines and reflects germarium length. Scale bar: 20 µm.

Previous results showed that, in the cap cells of the fly germarium, Dlp and Mmp2 genetically interact to restrict long-range Wg spreading (Wang and Page-McCaw, 2014). Further, Wg spreading was increased by the loss of *Mmp2* (Wang and Page-McCaw, 2014). Here, we tested how Mmp2 affects Wg spreading using gain-of-function and loss-of-function genetics. We found that *Mmp2* overexpression shortened the extracellular Wg gradient, effectively decreasing Wg spreading from its cap-cell source (Figure 5, A–C). Further, we found that overexpression of *Mmp2* shortened the length of the germarium as assayed by the Fascilin III (FasIII) staining, which detects the 2a/2b boundary (anatomical location of FSCs; Figure 5, A’–C’). Even when normalized to the decreased length of the germarium, the Wg spreading was decreased when *Mmp2* was overexpressed (Figure 5, A’’–C’’). These data are consistent with our cell culture data that cleaved Dlp sequesters more Wnts and promotes their internalization.

Reciprocally, decreasing levels of *Mmp2* (using two different allelic combinations) significantly extended the length of the germarium but did not significantly increase the Wg gradient (Figure 5, D–F’). When the Wg spread was normalized to the extended germarium length, it was stable (Figure 5, D’’–F’’) even though decreasing levels of *Mmp2* increase Wg signaling in the follicle stem cells, as evidenced by an accumulation of stalk cells (Wang and Page-McCaw, 2014). The location of the 2a/2b boundary is determined by gradients of multiple signaling pathways, including Wg (Vied *et al.*, 2012), and so the extension of the boundary may be more indirect evidence that Wg signaling is increased in the absence of Mmp2.

Our results here indicate that Dlp cleavage leads to Wg/Wnt sequestration and loss-of-function. Mmp2-mediated Wg sequestration and inhibition are consistent with the *Mmp2* mutant phenotypes of increased Wg signaling and the increased total level of Wg protein (Wang and Page-McCaw, 2014). It is also consistent with our data here that overexpression of *Mmp2* decreases the total spread of Wg from the cap cells (Figure 5, A–C). We propose a model where Dlp cleavage by Mmp2 results in increased Wg binding to cleaved Dlp, which is internalized, removing the cleaved Dlp-Wg complex from the extracellular signaling environment. Thus, Mmp2 provides a molecular brake on extracellular Wg availability. In contrast, uncleaved Dlp functions to distribute extracellular Wg (Baeg *et al.*, 2001; Baeg *et al.*, 2004; Kirkpatrick *et al.*, 2004; Kreuger *et al.*, 2004; Franch-Marro *et al.*, 2005; Han *et al.*, 2005; Wang and Page-McCaw, 2014), and Mmp2 decreases the fraction of uncleaved Dlp available on the cell surface for extracellular Wg spreading. Thus, proper Wg/Wnt distribution is achieved by coordinated activities of cleaved and uncleaved Dlp in the germarium.

## MATERIALS AND METHODS

Request a protocol through *Bio-protocol*

### Fly stocks

Fly stocks were cultured at 25°C on standard cornmeal, molasses, and yeast media. The stocks used in this study are described in Flybase. *TubP-Gal80^ts^* (BL7017) was obtained from the Bloomington *Drosophila* stock center. *SVS-dlp* (*PBac*{*602.P.SVS-1*}*dlpCPTI000445* [115031]) protein trap line was obtained from Kyoto Stock Center (Lowe *et al.*, 2014). In *SVS-dlp* flies, the Dlp genomic locus has an SVS-containing exon inserted into it, so that SVS-tagged *dlp* is expressed in the endogenous spatial and temporal patterns and at the endogenous levels (Delmore and Waghmare, 2025). Other stocks used include *w^1118^*, *C587-Gal4* (from Daniela Drummond-Barbosa), *UAS-Mmp2*, *Mmp2^Y53N^* temperature sensitive allele, *Mmp2^218ss^*, and Mmp2^W207*^. The *Mmp2* loss-of-function alleles have been previously described (Page-McCaw *et al.*, 2003; Wang and Page-McCaw, 2014). The UAS-mediated *Mmp2* expression was inhibited during development using *C587-Gal4*/*Gal80^ts^* by rearing the crosses at 18°C and allowing their progenies to develop at 18°C. One-day-old female flies of the appropriate genotype were collected, crossed with *w^1118^* males, and incubated at 29°C in a vial containing yeast paste for one day to allow *Mmp2* expression to be strongly in escort cells and weakly in follicle stem cells. Conditional expression of *Mmp2* for more than one day caused lethality. Conditional decrease of *Mmp2* levels was achieved by crossing temperature-sensitive *Mmp2^Y53N^*-mutant flies with *Mmp2^218ss^* or Mmp2^W207*^-mutant flies. The cross was reared at 18°C, and the progenies were allowed to eclose at 18°C. One-day-old females were collected, crossed with *w^1118^* males, and incubated at 29°C in a vial containing yeast paste for 7 days to decrease *Mmp2* levels, as a conditional decrease of *Mmp2* levels for 7 days generated a strong phenotype (Wang and Page-McCaw, 2014). Flies were transferred to fresh vials containing yeast paste every 2–3 d during the 7-day incubation.

### Cell culture and transfection

S2R+ cells were obtained from the *Drosophila* Genomics Resource Center and cultured in Schneider's medium supplemented with 10% heat-inactivated fetal bovine serum (Biowest) and 100 U/ml penicillin/streptomycin (Life Technologies). 2.5 × 10^5^ cells per well were plated 1 d before transfection either directly in 24-well plates (Figures 1–5; Supplemental Figures S2-S5) or on coverslips placed in the wells. Cells were transfected using CaCl_2_ method, induced using 700 µM CuSO_4_, and allowed to express protein at 18°C for 4 days as previously described (Wang and Page-McCaw, 2014). Following the 4-day incubation, cells were harvested for labeling with cell-impermeable SNAP substrate, immunohistochemistry, coimmunoprecipitation, or Western blot analysis.

Plasmids used in this study have been previously described and include *Metallothionein (MT)-Gal4* (for copper-inducible expression of Gal4), *UAS* (control), *UAS-SVS-dlp* (Waghmare *et al.*, 2020), *UAS-Mmp2-FLAG* (Wang and Page-McCaw, 2014), *UAS-Timp* (Page-McCaw *et al.*, 2003), *pAc5.1-wg-3XHA*, *pAc5.1-Wnt2–3XHA*, *UAS-Wnt4-3XFLAG*, and *pAc5.1-Wnt6–3XHA*, and *pMK33-wg*. *UAS-SVS-dlp* was generated by inserting the SVS tag near the N-terminus of Dlp by copying the homozygous-viable protein-trapped endogenous allele (Waghmare *et al.*, 2020). Of note, the previously used GFP-tagged Dlp construct contained GFP insertion in place of G69, whereas the SVS tag in SVS-Dlp is inserted after N103. (Lowe *et al.*, 2014; Wang and Page-McCaw, 2014; Waghmare *et al.*, 2020). *UAS-SNAP-dlp* was generated by replacing the Venus sequence in *UAS-SVS-dlp* with the SNAP sequence, which was copied from the pSNAP_f_ vector (NEB N9183S). The pSNAP_f_ vector sequence is available at FASTA HYPERLINK “https://www.neb.com/en-us/-/media/nebus/page-images/tools-and-resources/interactive-tools/dna-sequences-and-maps/text-documents/psnapfgbk.txt?rev=d75fc767bb9e4d8e951a54b1d941ecfc&hash = B576AB047A7746DB76D8E17FA5622C13” HYPERLINK “https://www.neb.com/en-us/-/media/nebus/page-images/tools-and-resources/interactive-tools/dna-sequences-and-maps/text-documents/psnapfgbk.txt?rev=d75fc767bb9e4d8e951a54b1d941ecfc&hash=B576AB047A7746DB76D8E17FA5622C13”GenBank. All HA-tagged Wnt plasmids are downstream of the actin enhancer and were obtained from K. Basler (Herr and Basler, 2012). *pMK33-wg* (Yanagawa *et al.*, 1998) was obtained from N. Perrimon. *UAS-Wnt4-3XFLAG* plasmid was obtained from M. Buszczak (Mottier-Pavie *et al.*, 2016). All plasmids used in this study were verified by sequencing. To minimize experimental variation, all cells were transfected with *MT-Gal4* and were incubated with CuSO_4_-containing media irrespective of whether or not Gal4 or CuSO_4_-mediated induction was required. Further, to minimize variation in the transfection efficiency, the total amount of DNA transfected in each well was kept constant by cotransfecting with the *UAS* (empty) vector as needed.

### Immunohistochemistry and labeling of cell-surface Dlp

Ovaries were stained for Dlp (to detect C-terminal polypeptide epitope amino acids 523-702) and GFP (to detect SVS epitopes) or extracellular Wg and FasIII. Mouse anti-Dlp (13G8; used 1:5), mouse anti-Wg (4D4; used 1:3), and mouse anti-FasIII (7G10; used 1:8) antibodies were obtained from Developmental Studies Hybridoma Bank (DSHB), and mouse anti-GFP antibody was obtained from UC Davis/National Institutes of Health NeuroMab Facility (clone N86/38, used 1:5). Extracellular Wg staining was performed as previously described (Strigini and Cohen, 2000; Wang and Page-McCaw, 2014) by dissecting the ovaries in ice-cold PBS (phosphate buffered saline) and incubating them with mouse anti-Wg antibody diluted in nonpermeabilizing blocking buffer (5% normal goat serum in PBS) on ice for 30 min. Samples were washed three times for 1 minute each with ice-cold PBS, fixed in 4% paraformaldehyde (Ted Pella) in PBS for 20 min at room temperature, and permeabilized and blocked using 5% normal goat serum in PBST (PBS plus 0.1% Triton X-100) for 1 h at room temperature. Samples were incubated with mouse anti-FasIII antibody for 1 h at room temperature and subsequently with Cy3-conjugated goat anti-mouse IgG1 (Jackson Immunoresearch 115–165-205), FITC-conjugated goat anti-mouse IgG2a (Jackson Immunoresearch 115–095-206) secondary antibodies (1:500, Jackson Immunoresearch) for 1 h in the dark at room temperature. Nuclei were stained using DAPI (Invitrogen) in PBST at a final concentration of 1 µg/ml for 10 min at room temperature in the dark, and the samples were mounted in Vectashield (Vector Laboratories). For anti-Dlp and anti-GFP stainings, a similar protocol was followed except that the primary antibodies were added after fixation and incubated overnight at 4°C. DAPI staining of nuclei in Figures 2, 3, and 5 is shown in blue.

Transfected S2R+ cells were fixed in 4% paraformaldehyde made in PBS and stained using a protocol previously described (Broderick *et al.*, 2012). DAPI-containing Vectashield was used to stain the nuclei in Figures 2 and 3. Primary antibodies used: mouse anti-GFP (clone N86/38, 1:25; UC Davis/National Institutes of Health NeuroMab Facility), mouse anti-Dlp (13G8, DHSB, 1:50). Secondary antibodies (from Jackson Immuno Research) used were: FITC-conjugated goat anti-mouse IgG2a and Cy3-conjugated goat anti-mouse IgG1 (1:250). Permeabilizing and nonpermeabilizing conditions during the staining process were maintained by using PBST or PBS, respectively.

Cell surface labeling of Dlp in *UAS-SNAP-dlp* or *UAS-SNAP-dlp+Mmp2* transfected cells was done by incubating the cells on ice with cell impermeable SNAP-Surface 549 red fluorescent substrate (NEB S9112S, Figure 2) or SNAP-Surface 488 green fluorescent substrate (NEB S9124S; Supplemental Figure S3) following the standard transfection protocol described above. Following 3X washes with ice-cold PBS, cells were incubated with prewarmed media at 25°C for 10 min or 20 min to allow internalization, following which they were fixed with 4% paraformaldehyde. Samples were washed with PBS and mounted in DAPI-containing Vectashield. We note that in the SNAP-labeling experiments, in the presence of Mmp2, the cells appeared more flattened and bigger compared with similarly transfected cells in the antibody staining experiments. We speculate that this change in morphology is likely because, in the SNAP-labeling experiments, the cells were cultured on coverslips, which were flipped and mounted on the slide post labeling. In contrast, in our antibody staining experiments, cells were plated in the culture wells directly, resuspended post-transfection, and allowed to adhere to multi-well glass slides before fixing and antibody staining.

### Coculture of transfected cells

Cells were transfected with *pUAST* (control), *UAS-SVS-dlp* with or without *UAS-Mmp2-FLAG,* or *UAS-Timp* and induced for two days using CuSO_4_ as described above; cotransfection with *UAS-SVS-dlp* and *UAS-Mmp2-FLAG* was done in duplicate. After two days, the media was removed from *pUAST*, *UAS-SVS-dlp*, and *UAS-SVS-dlp*+*UAS-Mmp2-FLAG*–transfected cells, and cells and conditioned media from *pUAST*-transfected cells were added to *pUAST*, *UAS-SVS-dlp*, and one batch of *UAS-SVS-dlp*+*UAS-Mmp2-FLAG*–transfected cells and cocultured for two more days. Additionally, a separate well containing *UAS-SVS-dlp*+*UAS-Mmp2-FLAG*–transfected cells was cocultured with *Timp*-expressing cells and cocultured for two more days, following which cells were harvested either for Western blot or immunohistochemistry. Cells and conditioned media from two wells expressing *pUAST* or *UAS-Timp* were added to each of the appropriate wells.

### Co-immunoprecipitation and Western blots

Co-immunoprecipitation assay and Western blots were performed as previously described (Waghmare *et al.*, 2020). Briefly, cells were harvested by centrifugation and lysed in protease inhibitor (Halt Protease inhibitor single use cocktail, Thermo Scientific) containing lysis buffer 2 mM Tris HCl, 150 mM NaCl, 1% NP-40 substitute, 1 mM EDTA, 5% glycerol, pH:7.4 lysis buffer. The lysate was clarified to remove debris by centrifuging samples at 13,000 rpm for 5 min at 4°C, and the clarified lysate was diluted four times with lysis buffer before incubating with GFP-Trap Magnetic Agarose beads (Chromotek) for 30 min at 4°C. For immunoprecipitation of conditioned media, an equal amount of media was incubated with GFP-Trap magnetic agarose beads. Following 3 × 30 min washes with lysis buffer after incubation, the beads were incubated with 1X reducing sample buffer (1 × NuPage LDS sample buffer + 5% beta-mercaptoethanol or 1X Laemmli sample buffer + dithiothreitol) and heated at 90°C for 5 min to elute protein complexes. SDS-PAGE was performed on eluted samples, followed by Western blotting.

SDS-PAGE and Western blots shown in all figures are from experiments that were done under reducing conditions, with the exception of the blot shown in Figure 1B, which was done under nonreducing conditions. Reducing conditions are described above, and non-reducing conditions were maintained by using 1 × NuPage LDS sample buffer (Invitrogen) without a reducing agent. Transfected cells were harvested by centrifugation, washed with 1X PBS, and lysed in either reducing or nonreducing sample buffers. Following denaturation of samples by heating at 90°C for 2 min, precast Tris-glycine (10% Mini-PROTEAN TGX) gels (Bio-Rad Laboratories) were used to separate proteins by SDS–PAGE. Wet-transfer protocol was used to transfer proteins onto Hybond-C Extra nitrocellulose membranes (GE Healthcare).

Immunoblotting was performed by incubating the membranes in primary antibodies overnight at 4°C, followed by incubation in secondary antibodies for 2 h at room temperature in the dark. The membranes were subject to 3 × 10 min washes with 0.1% PBT (1X PBS + 0.1% Tween-20), 1 × 5-min wash with 1X PBS, and scanned using Odyssey Infrared Imaging System (LI-COR Biosciences). Primary antibodies used for Western blots in this study include mouse anti-actin (MAB1501R, EMD Millipore, 1:2000), rabbit anti-GFP (Torrey Pines Biolabs; 1:2500), rat anti-HA (3F10, Roche, 1:1000), and mouse anti-FLAG M2 (F3165; Sigma-Aldrich). Secondary antibodies (1:5000) used in this study include: donkey anti-rabbit IgG conjugated to IRDye 680, and goat anti-rat IgG or goat anti-mouse IgG conjugated to IRDye 800CW (LI-COR Biosciences). In Figure 1A, blots were probed first with anti-GFP, stripped by incubating the blot in NewBlot Nitro Stripping buffer (LI-COR) for 10 min, and reprobed with anti-Dlp after washing with 0.1% PBT.

### Imaging, quantification, and statistics

Images were taken using Zeiss Apotome Imager M2 (Figures 2 and 3; Supplemental Figure S3) or Nikon Ti2 Eclipse (Figure 5). Figures were generated using Affinity Photo and Affinity Designer. All quantification was done in ImageJ/FIJI. Results reported in Figure 1, A and B, were replicated in >3 independent experiments, results reported in Figure 2, A–C, D, and E, and Figure 5, D were replicated in 2 independent experiments, and results reported in Figure 4 and Supplemental Figure S4 were replicated in 3 independent experiments per Wnt. Additional blots showing replicates of data shown in Figure 4 and Supplemental Figure S4 are shown in Supplemental Figure S5. In Figures 2, F and G, and Supplemental Figure S3, only cells where cell surface labeling of Dlp was visible to the eye were analyzed. Images used for intensity measurements, counting punctae in Figure 2, F and G, and measurement of Wg spread relative to the size of the germarium were acquired under identical conditions for comparison across samples in each experiment. Sum Z-stack was used to analyze Dlp punctae using “analyze particles” in Figure 2, F and G. A constant punctae size threshold was applied across all samples analyzed. Although some Z-stacks were used for punctae analysis, the signal from the cell-surface labeling did not significantly interfere with our analysis because this signal was largely nonpunctate and therefore excluded by the punctae size threshold applied in the analysis. In Supplemental Figure S3, single slices from the middle region of cells were used to manually determine whether cells contained intracellular punctae for quantification. Sum Z-stack was used to calculate the average signal intensity within cells or tissue by outlining cells or tissue, respectively.

Wg spread was measured by drawing a line from the cap cells to the endpoint of the continuous visible Wg gradient (as indicated by arrows in representative images) in sum Z stack. Wg gradient was determined by eye by focusing on the edge of the germarium on each side. In samples where the Wg spread was uneven along the edges of the germarium, the side with a longer spread was used to determine the spread. The location of the 2a/2b boundary was determined by drawing a line from the cap cells to the 2a/2b boundary, detected by the FasIII staining in the sum Z stack. All samples were scored blindly to the identity of the samples.

For calculating fold change in Wnt pull down with uncleaved and cleaved Dlp, average band intensities of N-terminal SVS-Dlp and Wnts were measured in ImageJ. Ratio of Wnt intensity/uncleaved Dlp intensity in the absence of Mmp2 was used to estimate how much Wnt was pulled down by the uncleaved Dlp or cleaved Dlp in the presence of Mmp2.

Statistical analyses were done using GraphPad Prism. Mean and standard error of mean (SEM) are represented on the dot plots in Figures 2, 3, and 5. Student's *t* test was used in Figures 2, 3, and 5, and chi-square test was done to determine differences in frequencies for each category within the indicated samples in Figure 2 (*p = 0.05; **p < 0.01; ***p < 0.001; n.s.: not statistically significant; n: sample size; AU: arbitrary units).

## Supporting information





## Abbreviations used:

ADAM17: a disintegrin and metalloproteinase 17
A-P: anterior-posterior
CpC: cap cells
CRD: cysteine rich domain
CuSO_4_: copper II sulphate
Cy3: cyanine 3
DAPI: 4',6-diamidino-2-phenylindole
Dlp: Dally-like protein
DNA: deoxyribonucleic acid
DSHB: developmental studies hybridoma bank
EDTA: ethylenediaminetetraacetic acid
FasIII: Fasciclin III
FITC: Fluorescein isothiocyanate
FLAG: DYKDDDDK peptide tag
FSC: follicle stem cells
GAG: glycosaminoglycans
Gal4: galactose activator 4
Gal80^ts^: temperature-sensitive galactose 80
GFP: green fluorescent protein
HA: hemagglutinin tag
LDS: lithium dodecyl sulfate
Mmp2: matrix metalloproteinase 2 MT-Metallothionein
NaCl: sodium chloride
NP-40: nonidet P-40
PBS: phosphate buffered saline
PBST: phosphate buffered saline with TritonX-100
PBT: phosphate buffered saline with Tween20
SDS-PAGE: sodium dodecyl sulfate- polyacrylamide gel electrophoresis
SEM: Standard error of mean SNAP
tag: engineered version of the ubiquitous mammalian enzyme, encoded in humans by O-6-methylguanine-DNA methyltransferase
SVS: StrepII-Venus-StrepII
Timp: tissue inhibitor of metalloproteinase Tris
HCl: tris(hydroxymethyl)aminomethane hydrochloride
UAS: upstream activating sequence
Wg: wingless
Wnt: wingless+ Int (a mouse proto-oncogene).
