# Supplementary material for: Matrix metalloproteinase 2 destabilizes Dally-like protein to restrict extracellular Wingless distribution
Source: Mol Biol Cell. 2025 Oct 15;36(12):br32. doi: 10.1091/mbc.E22-09-0434 (PMC12636523; doi:10.1091/mbc.E22-09-0434)
Supplement: Supplementary file 1 [file mbc-36-br32-s001.pdf]

Supplemental Materials

Molecular Biology of the Cell

Waghmare *et al*.

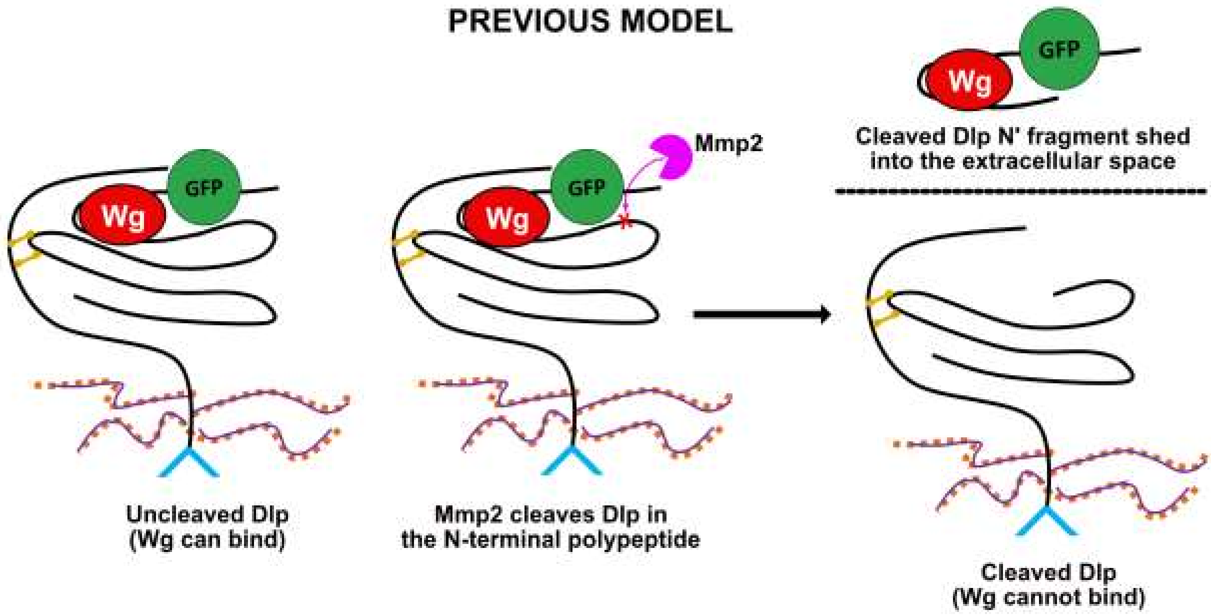
PREVIOUS MODEL
GFP
Wg
Cleaved Dlp N' fragment shed
into the extracellular space
GFP
Wg
Mmp2
GFP
Wg
Uncleaved Dlp
(Wg can bind)
Mmp2 cleaves Dlp in
the N-terminal polypeptide
Cleaved Dlp
(Wg cannot bind)

Fig. S1: Previous model of the effect of Mmp2 cleavage on Dlp structure and function.
Uncleaved Dlp binds Wg to facilitate long-range Wg distribution, whereas cleaved Dlp cannot bind Wg, attenuating the long-range distribution of Wg.

A

MLHQQQQQH LHCRRKATAT TTARLVIFSS PLLLLLLTTH LPPTLQADNG PAPQVAALAA
PNPAGGVAGS SIIDQFSPNC SAVTHIFQAR GIDAIEIPQK PSNAHHHHHH GWSHPQFEKG
AVSKGEELFT GVVPILVELD GDVNGHKFSV SGEGEGDATY GKLTLKLICT TGKLPVPWPT
LVTTLGYGLQ CFARYPDHMK QHDFFKSAMP EGYVQERTIF FKDDGNYKTR AEVKFEGDTL
VNRIELKGID FKEDGNILGH KLEYNYNSHN VYITADKQKN GIKANFKIRH NIEDGGVQLA
DHYQQNTPIG DGPVLLPDNH YLSYQSALSK DPNEKRDHMV LLEFVTAAGI TLGMDELYKG
AWSHPQFEKS TRERVLRYCE SPSVGTCCTY NMETRMAMQS RQQLEGHTKD QISRMSGILG
SKATKFKDIF TALLKESRTQ FNSMFIRTYG VIYERNSYVF SDLFKELETY FANGRVDLLE
VMDKFFNTLY QKMFTVLNTQ YTFDENYMRC VSEHMKELKP FGDVPDKLSV QIKRSFVATR
TYGQALTTAS EVAKKVLNVR LNADCTGALT KMQHCGACKG YTEKPCTNYC VNVIKGCLHY
QHEFDSEWEN FAMAMDKVAE RLLGSFNIVM VVEPLNIKIS EAIMNFQDSG QDITNRVFQG
CGRPKLKKMK RSISPKLQGV QILNARSPVE ADTLDIDETL DEAIVLRERR AAEPGSQETS
AQQSQEQGVG KSGNGGGGGG GNNRRQQQRR KQQQQRRKQQ NNRDDNDDDD NESGGGREPI
LDRIVRDIRQ RVKDYKKFWS NLPHSVCSNE DIASSSDVDG MCWNGHTIDR YMHSITTEHG
SNPEFTGNPA STKQTAQMAS QLSHLKNAIV HLRNAYNGQD VEWSEQEELP YAGSGAGSGS
GSEDDEDDDE GSGLGPFEPS HKPDVERPSV DADNDDDEDA GGRGHMPTHT SRPTSGVDDK
NPLIHTTHFD QDHNDLDEDH RQLDEDEDTD AGHDGANDNR SSDAPEKMTL RRALVVYLLP
LYMAWFGGVC ADLL*

B IP: GFP from conditioned media (reducing conditions)

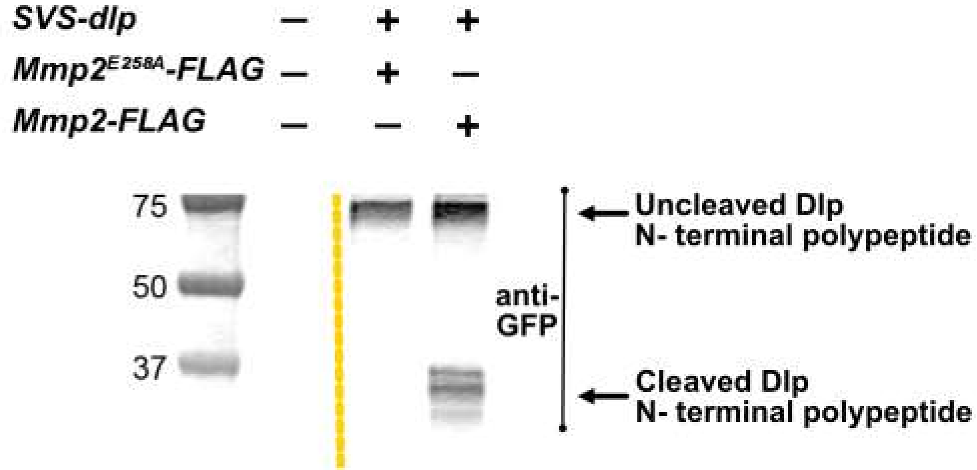

Fig. S2: Mapping cysteines in Dlp suggests that the cleaved N-terminal Dlp polypeptide is released only under reducing conditions.
(A) Signal peptide (grey, italicized), position of SVS tag in SVS-Dlp (green), estimated cleavage site (underlined, gray highlight) based on the size of the cleaved fragments on reducing western blot, seven disulfide bonds (dotted lines) are shown. Four of the seven disulfide bonds (light and dark blue dotted lines) including the two disulfide bonds linking the N-terminal and C-terminal polypeptides (dark blue dotted lines) were previously reported (Kim *et al.*, 2011), other disulfide bonds (pink and brown dotted lines) including the one that holds the cleaved fragments seen on the reducing blot together (brown dotted line) can be predicted based on the more recent reported structure of Dlp (McGough *et al.*, 2020) using UCSF ChimeraX (Pettersen *et al.*, 2021).
(B) Conditioned media from cells transfected with indicated plasmids was immunoprecipitated using anti-GFP beads and the immunoblot was probed with anti-GFP. The dotted yellow line indicates that the intervening lane on the blot was excluded.

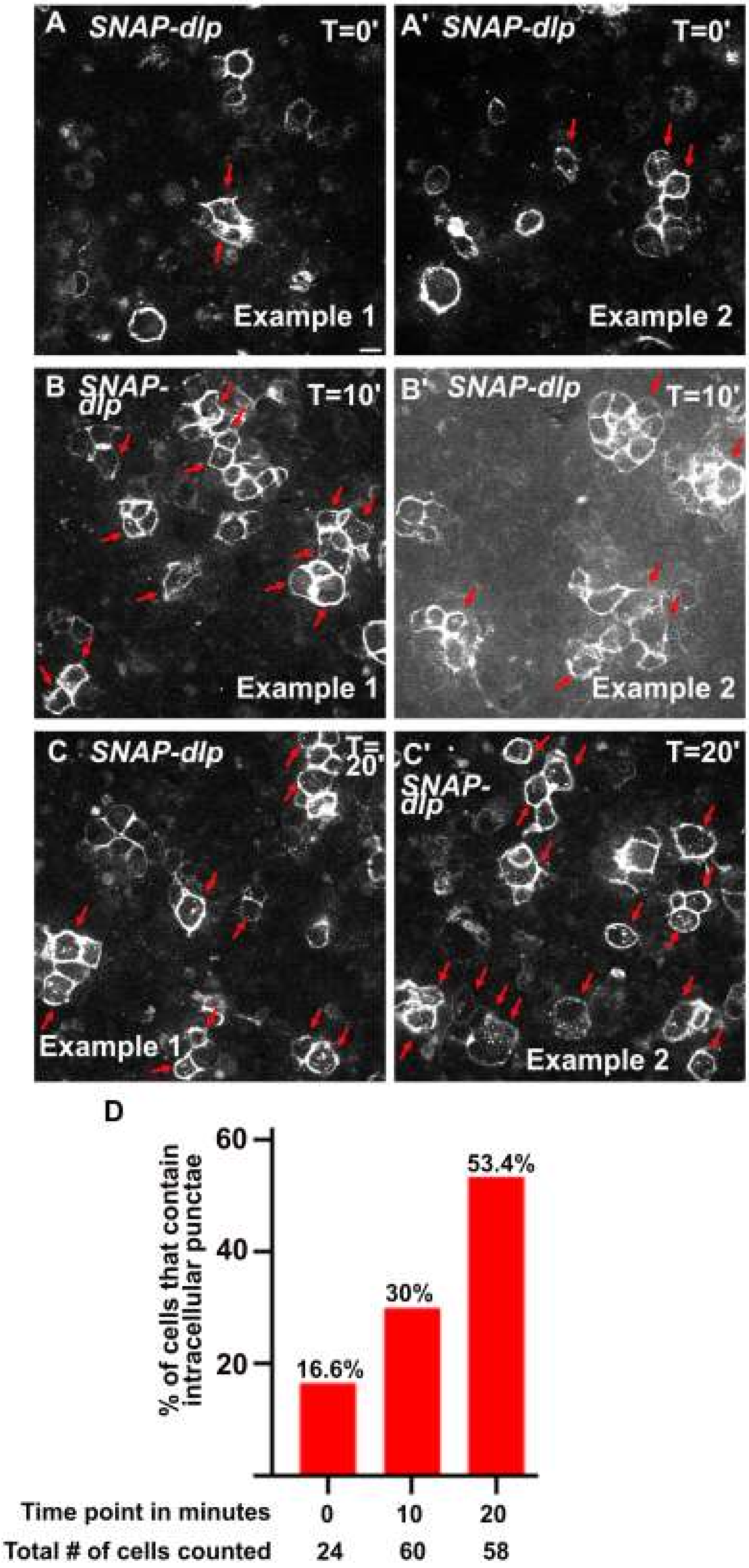
A SNAP-dlp T=0'
Example 1
A' SNAP-dlp T=0'
Example 2
B SNAP-dlp T=10'
Example 1
B' SNAP-dlp T=10'
Example 2
C SNAP-dlp T=20'
Example 1
C' SNAP-dlp T=20'
Example 2
D
% of cells that contain intracellular punctae
60
40
20
16.6%
30%
53.4%
Time point in minutes 0 10 20
Total # of cells counted 24 60 58

Fig. S3: Validation of the cell surface labeling of Dlp and its internalization using *SNAP-dlp* construct.

(A-C) *SNAP-dlp* transfected cells were incubated with cell-impermeable SNAP-Surface 488 fluorogenic substrate (to label cell-surface Dlp) on ice. Following labeling, cells were fixed immediately (A, A'), or incubated at 25° for 10 minutes (B, B') or 20 minutes (C, C') to allow internalization. Optical sections of fixed cells were obtained. Red arrows show cells with intracellular punctae. Cells shown in A, A' were imaged at 2.5X higher exposure than cells shown in B-C'. Scale bar: 10 µm

(D) Quantification of data shown in A-C. Single slices shown above were used to determine whether cells contained internalized punctae for quantification.

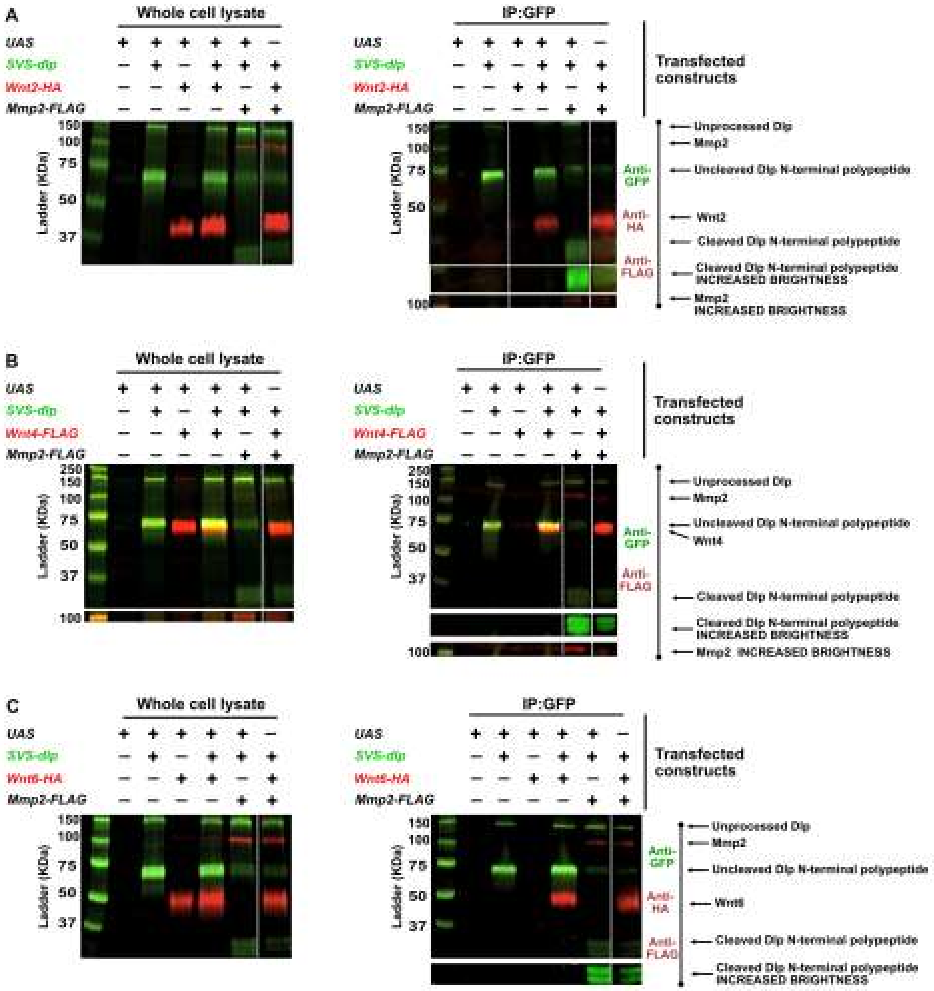
A
Whole cell lysate
IP:GFP
UAS
SVS-dlp
Wnt2-HA
Mmp2-FLAG
Transfected constructs
Ladder (KDa)
150
100
75
50
37
Anti-GFP
Anti-HA
Anti-FLAG
Unprocessed Dlp
Mmp2
Uncleaved Dlp N-terminal polypeptide
Wnt2
Cleaved Dlp N-terminal polypeptide
Cleaved Dlp N-terminal polypeptide INCREASED BRIGHTNESS
Mmp2 INCREASED BRIGHTNESS
B
Whole cell lysate
IP:GFP
UAS
SVS-dlp
Wnt4-FLAG
Mmp2-FLAG
Transfected constructs
Ladder (KDa)
250
150
100
75
50
37
Anti-GFP
Anti-FLAG
Unprocessed Dlp
Mmp2
Uncleaved Dlp N-terminal polypeptide
Wnt4
Cleaved Dlp N-terminal polypeptide
Cleaved Dlp N-terminal polypeptide INCREASED BRIGHTNESS
Mmp2 INCREASED BRIGHTNESS
C
Whole cell lysate
IP:GFP
UAS
SVS-dlp
Wnt6-HA
Mmp2-FLAG
Transfected constructs
Ladder (KDa)
150
100
75
50
37
Anti-GFP
Anti-HA
Anti-FLAG
Unprocessed Dlp
Mmp2
Uncleaved Dlp N-terminal polypeptide
Wnt6
Cleaved Dlp N-terminal polypeptide
Cleaved Dlp N-terminal polypeptide INCREASED BRIGHTNESS

Fig. S4: Despite lower levels of cleaved Dlp, both uncleaved and cleaved SVS-Dlp pull down comparable amounts of tagged Wnt2, Wnt4, or Wnt6 suggesting that cleaved Dlp exhibits higher affinity for Wnts.

(A-C) Western blots of **(Left)** whole cell lysates or **(Right)** anti-GFP immunoprecipitated preps probed with anti-GFP (green), anti-HA (red), and anti-FLAG (red) antibodies. The anti-GFP recognizes N-terminal SVS-Dlp. The anti-HA recognizes 3XHA-tagged Wnt2 and Wnt6, and the anti-FLAG recognizes 3XFLAG-tagged Wnt4 and FLAG-tagged Mmp2. Vertical white dotted lines indicate that the intervening lanes were excluded.

Biological replicate 2

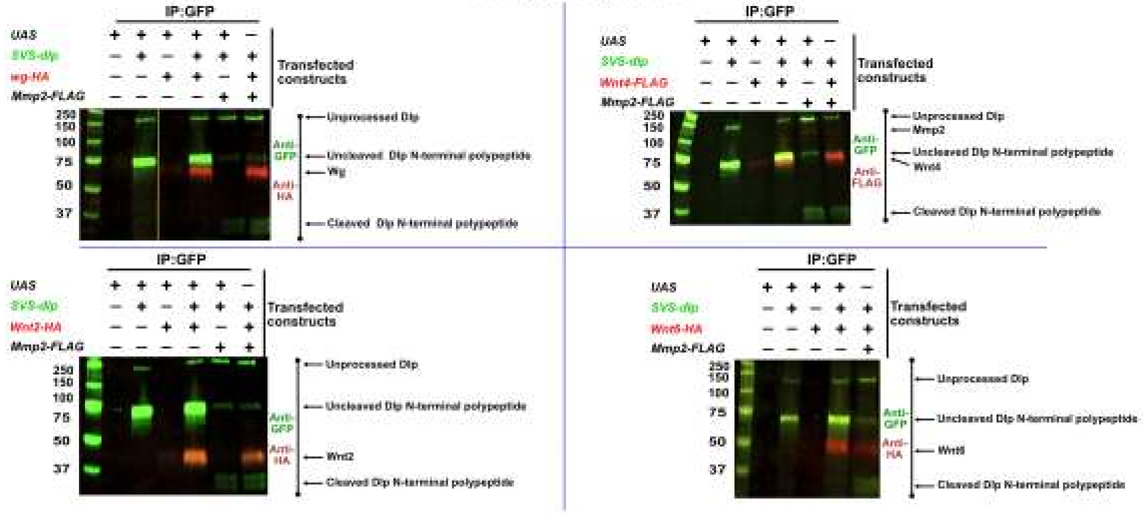

Biological replicate 3

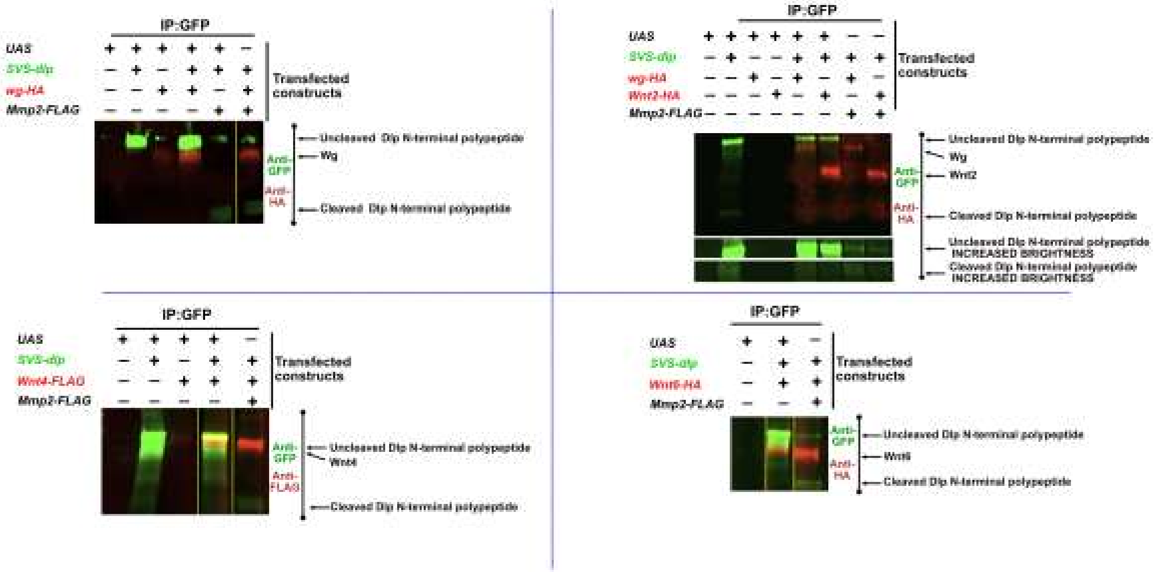

Fig. S5: Biological replicates showing that cleaved Dlp sequesters more Wnts
Western blots of anti-GFP immunoprecipitated samples from lysates of cells with indicated plamids are shown. Samples were reduced, and blots were probed with indicated antibodies. Parts of data shown in IP blots in replicate 2 (Wnt2, Wnt4, and Wnt6) have been previously published (Waghmare *et al.*, 2020).
